# Cholera

**Published:** 1866-02

**Authors:** Charles A. Lee


### Correspondence.

### CHOLERA.

To the Editor of the Buffalo Medical and Surgical Journal ;

I enclose you the following communication, just received from Dr. Marsden, of Quebec, being a "*Plan of Quarantine for Cholera,*" for that city, prepared by him and approved by a committee of the medical profession to whom the subject was referred. You will, I think, confer a great favor on the profession generally, as well as the municipal authorities of this city, by giving it a place in your pages. You will perceive that the whole plan is founded on the principle that the Asiatic cholera is a *portable, controllable* and *communicable* disease:

This is the same view which I took recently, as you are aware, in my letter to Dr. Sayre, Health Commissioner of New York, and published in the daily papers. In justice to myself, perhaps, I ought to state that I did not intend to claim the theory and views set forth in that communication *as original with myself,* although long entertained by me, in common with many others. I have studied the history of cholera very closely, since its first introduction into our country in 1832, when I had charge of one of the largest cholera hospitals in New York city, and I gradually came to the conclusion, that though not contagious, in the ordinary sense of the word, it was nevertheless *portable* in some way, and in all probability through the dejections of those affected with it, whether in the form of *cholerine* or an acute attack. In my edition of Copland's "Dictionary of Practical Medicine," published many years ago, I advocated, in common with the author, the doctrine of *contingent contagiousness* of the disease; had the word *infection* been substituted for that of *contagion*, it would have come nearer the truth, inasmuch as we can no longer doubt that cholera may not only be transmitted by *persons* but also by *effects*.

I think the profession, generally, in our country, as well as abroad, have been gradually adopting the same conclusions. The principal physicians of Germany, for example, have during the last ten years advocated the theory of the transmission of the disease by the choleraic dejections of persons affected with it, among whom may be named Professors Pettenkofer, Niemayer, Dielbruck and others. Notices of the same theory have appeared in several of the foreign medical journals during the last year, and also in the *Buffalo Medical and Surgical Journal* of December, 1865, in a very able article by Prof. Niemayer of Tubingen, translated from the German by Dr. T. A. McGraw, of Detroit. This paper probably contains the fullest and most satisfactory exposition of this doctrine yet given to the profession in our country; and had I been writing an article for a medical journal, I should have taken great pleasure in referring to this paper, and given my friend Dr. McGraw full credit for his very faithful and admirable translation of the same. It is also no more than justice to my respected colleague, Prof. Rochester, to state, that he has, for many years past entertained precisely the same views in regard to the mode of propagation of the disease, and has taught them in his annual courses of lectures in the Medical Department of the Buffalo University.

CHARLES A. LEE, M. D.

